# Paradoxical Rise in Pulmonary Artery Pressure Following Successful Mavacamten Therapy for Obstructive Hypertrophic Cardiomyopathy: A Case Report

**DOI:** 10.1002/ccr3.72859

**Published:** 2026-06-04

**Authors:** Mengdian Zhang

**Affiliations:** ^1^ Department of General Practice, Yuetan Community Health Center, Fuxing Hospital Capital Medical University Beijing China

**Keywords:** aged, cardiomyopathy, hypertrophic obstructive, catheterization, swan‐Ganz, hypertension, pulmonary

## Abstract

A paradoxical rise in pulmonary artery pressure on follow‐up echocardiography after successful mavacamten therapy for obstructive hypertrophic cardiomyopathy (oHCM) prompted right heart catheterization, which demonstrated combined pre‐ and post‐capillary pulmonary hypertension and guided the decision to continue mavacamten while withholding targeted pulmonary vasodilator therapy.

## 1 Introduction

Mavacamten, a cardiac myosin inhibitor, effectively reduces left ventricular outflow tract obstruction and improves symptoms in patients with oHCM, but also affects pulmonary artery pressure [1, 2, 3]. However, a paradoxical rise in pulmonary artery systolic pressure following successful gradient reduction with mavacamten has rarely been documented.

OHCM is itself frequently complicated by pulmonary hypertension (PH), with a reported prevalence ranging from 12.6% to 51% [4, 5, 6]. In this setting, PH is most commonly post‐capillary (Group 2), driven by elevated left ventricular filling pressures. Group 2 PH is further classified into isolated post‐capillary PH (IpcPH) and combined pre‐ and post‐capillary PH (CpcPH); current guidelines recommend against the use of pulmonary vasodilator therapy in this population, regardless of hemodynamic subtype [7]. When pulmonary artery pressure rises during mavacamten therapy, however, the presence of a pre‐capillary component raises the possibility that the PH may be driven at least in part by primary pulmonary vascular disease—for which targeted therapy could be indicated. Conversely, treating such a patient empirically with pulmonary vasodilators in the setting of elevated left ventricular filling pressures could precipitate acute pulmonary edema. Nevertheless, invasive hemodynamic confirmation of CpcPH in this specific clinical context, particularly in very elderly patients, is scarcely reported, making this distinction especially challenging. This tension underscores the need for invasive hemodynamic assessment to guide therapy.

We report the case of an 80‐year‐old woman with oHCM in whom successful LVOT gradient reduction with mavacamten was accompanied by a paradoxical rise in pulmonary artery systolic pressure. Right heart catheterization revealed CpcPH, confirming that the PH remained driven by left heart disease despite the elevated pulmonary pressure. This finding directly informed the decision to continue mavacamten and withhold targeted pulmonary vasodilator therapy, illustrating the importance of invasive hemodynamic classification when a discordant treatment response is observed. This case also adds to the limited real‐world data on mavacamten in very elderly patients, a population in whom treatment‐related hemodynamic responses are not well described.

## 2 Case History and Examination

An 80‐year‐old woman presented with a 6‐month history of exertional chest tightness, dizziness, fatigue, and low mood, with progressive bilateral lower limb edema for the preceding 2 months. The chest tightness was substernal, occurred predictably during moderate exertion, and resolved with rest. She reported no syncope, palpitations, or orthopnea. She had no prior diagnosis of coronary artery disease.

Her past medical history included hypertension. Her sister had been diagnosed with oHCM, with no reported history of syncope or sudden death.

On examination, her blood pressure was 139/84 mmHg (heart rate 78 beats per minute) and oxygen saturation 96% on room air. She appeared chronically ill, with pale conjunctivae. No jugular venous distension or hepatojugular reflux was noted. A systolic murmur was audible at the left sternal border (3rd–4th intercostal space), which became louder when moving from a supine to a seated position. Bilateral pitting edema (2+–3+) was present, with calf circumference measuring 27–30 cm.

## 3 Differential Diagnosis, Investigations, and Treatment

Initial investigations showed microcytic anemia (hemoglobin 90 g/L), normal biochemistry, and non‐specific ST‐T changes on electrocardiogram. Arterial blood gas analysis on room air revealed mild hypoxemia (PaO_2_ 72 mmHg), which was initially attributed to the patient's anemia and age‐related changes. Coronary computed tomography angiography was normal. N‐terminal pro‐brain natriuretic peptide (NT‐proBNP) was 1223 pg/mL, and the six‐minute walking distance was 317 m.

The patient was initially managed with metoprolol 25 mg twice daily and sublingual glyceryl trinitrate for suspected angina, and ferrous succinate 0.2 g twice daily for presumed iron deficiency anemia. Despite these interventions, her exertional chest tightness and dyspnea persisted, and her hemoglobin improved only modestly to 104 g/L, arguing against coronary artery disease and anemia‐related heart failure as the primary etiologies.

The combination of ongoing exertional chest tightness, persistent bilateral edema, and elevated NT‐proBNP in the absence of pulmonary congestion or orthopnea suggested right heart dysfunction without left ventricular failure. Differential diagnoses considered for the right heart dysfunction included pulmonary hypertension, whether primary pulmonary arterial hypertension or secondary to left heart disease, chronic thromboembolic disease, and sleep‐disordered breathing. Acute coronary syndrome and pulmonary embolism were considered less likely given the chronic, progressive course and the absence of electrocardiographic or biomarker evidence of acute ischemia. The systolic murmur that intensified with preload reduction raised particular suspicion for oHCM, which could account for both the exertional symptoms and, through elevated left ventricular filling pressures, secondary pulmonary hypertension.

Transthoracic echocardiography revealed asymmetric basal septal hypertrophy with a maximal wall thickness of 19 mm, systolic anterior motion (SAM) of the mitral valve, and a peak left ventricular outflow tract (LVOT) gradient of 97 mmHg at rest, confirming the diagnosis of oHCM. The initial echocardiogram also noted mild elevation of pulmonary artery systolic pressure (PASP), estimated at 35 mmHg. A 24‐h ambulatory electrocardiographic monitor showed sinus rhythm with occasional atrial premature complexes and no ventricular arrhythmias.

She was started on mavacamten, in addition to bisoprolol, sacubitril/valsartan, and empagliflozin.

## 4 Outcome and Follow‐Up

At a 2‐month follow‐up, she reported that exertional chest tightness still occurred with moderate exertion, though less frequently. Her hemoglobin had normalized to 124 g/L with continued iron supplementation. The bilateral lower limb edema had improved from 2+−3+ to 1+−2+, and calf circumference had decreased from 27–30 cm to 26–28 cm. Her six‐minute walking distance improved from 317 m to 414 m, and NT‐proBNP decreased from 1223 pg/mL to 454 pg/mL. Liver function tests remained within normal limits throughout treatment.

Repeat echocardiography showed a reduction in the peak LVOT gradient from 97 mmHg to 64 mmHg, with an unchanged left ventricular ejection fraction. However, the estimated PASP had increased from 35 mmHg to 41 mmHg. A follow‐up 24‐h ambulatory electrocardiographic monitor showed no atrial fibrillation or other arrhythmias.

The paradoxical dissociation between improving LVOT obstruction and rising pulmonary artery pressure, together with the incomplete resolution of symptoms and edema, prompted further hemodynamic evaluation. Right heart catheterization revealed a mean pulmonary artery pressure (mPAP) of 31 mmHg, a pulmonary capillary wedge pressure (PCWP) of 21 mmHg, and a pulmonary vascular resistance (PVR) of 2.9 Wood units (WU), confirming combined pre‐ and post‐capillary pulmonary hypertension (CpcPH; Group 2) secondary to left heart disease.

No pulmonary vasodilator therapy was initiated, and mavacamten was continued with close monitoring. The patient was advised to avoid strenuous physical activity, heavy lifting, and abrupt postural changes. A sleep study and ventilation/perfusion scintigraphy were planned to exclude contributing factors to PH. Regular cardiopulmonary follow‐up was arranged for serial monitoring of LVOT gradient, pulmonary artery pressure, and liver function. Given the autosomal dominant inheritance of oHCM and her sister's diagnosis, cascade screening of first‐degree relatives was recommended. The key investigations before and after mavacamten therapy are summarized in Table 1.

**TABLE 1:** Key investigations before and after mavacamten therapy.

Parameter	Before treatment	After 2 months of mavacamten	Change
Symptoms and functional status			
Exertional chest tightness	Present	Improved, though not fully resolved	Improved
Bilateral lower limb edema (pitting)	2+ to 3+	1+ to 2+	Improved
Calf circumference (cm)	27–30	26–28	Decreased
NYHA functional class	II	II	Unchanged
Six‐minute walking distance (m)	317	414	↑ 97
Echocardiography			
Peak LVOT gradient (mmHg)	97	64	↓ 33
Estimated PASP (mmHg)	35	41	↑ 6
E/e’ ratio	21.3	—^a^	—
Left atrial diameter (mm)	44	40	↓ 4
LVEF (%)	Normal^b^	70	—
Maximal septal wall thickness (mm)	19	19	Unchanged
SAM of the mitral valve	Present	Present	Unchanged
Right heart catheterization			
mPAP (mmHg)	—	31	—
PCWP (mmHg)	—	21	—
PVR (WU)	—	2.9	—
Laboratory investigations			
NT‐proBNP (pg/mL)	1223	454	↓ 769
Hemoglobin (g/L)	90	124	↑ 34
Liver function tests	Normal	Normal	Unchanged
Other investigations			
24‐h ambulatory ECG	Occasional atrial premature complexes	No atrial fibrillation or other arrhythmias	Improved
Arterial blood gas (PaO_2_, mmHg)	72	—	—
Oxygen saturation on room air (%)	96	98	↑ 2
Coronary CT angiography	Normal	—	—

Abbreviations: LVEF, left ventricular ejection fraction; LVOT, left ventricular outflow tract; mPAP, mean pulmonary artery pressure; NT‐proBNP, N‐terminal pro‐brain natriuretic peptide; NYHA, New York Heart Association; PASP, pulmonary artery systolic pressure; PCWP, pulmonary capillary wedge pressure; PVR, pulmonary vascular resistance; SAM, systolic anterior motion; WU, Wood units.

^a^ E/e’ was not reported numerically on the follow‐up study; the report noted qualitatively that the mitral annular diastolic tissue Doppler spectrum showed e’/a’ < 1.

^b^ Left ventricular ejection fraction was not explicitly reported on the initial study though left ventricular systolic function was described as grossly normal.

## 5 Discussion

The patient presented with exertional chest tightness commonly attributed to coronary artery disease, but the lack of relief with nitrates and metoprolol, a murmur that intensified upon sitting, bilateral edema without left ventricular systolic dysfunction, and a normal coronary CT angiography suggested an alternative etiology. Together with a positive family history, these findings pointed toward oHCM.

The dynamic systolic murmur was consistent with oHCM [1], and echocardiography confirmed the diagnosis. Two clinical features, however, remained unexplained: peripheral edema without left ventricular systolic dysfunction and persistent symptoms despite correction of anemia. These findings prompted re‐review of the initial echocardiogram, which identified mild elevation of PASP (35 mmHg) that had been attributed to left ventricular diastolic dysfunction and not regarded as requiring independent intervention.

Mavacamten was initiated, for which the patient met guideline‐directed indications [2]. An exploratory analysis of the EXPLORER‐HCM trial found that the benefits of mavacamten extended to older patients [8]. The present case supports this finding in an 80‐year‐old patient, although data specifically in octogenarians remain limited, as the mean age of trial participants was approximately 60 years [2]. A recent real‐world study of 2440 patients on mavacamten demonstrated that new‐onset atrial fibrillation or flutter occurred in 5% and new heart failure in 4% over a median follow‐up of 8 months [9], underscoring the importance of continued monitoring. At the 2‐month follow‐up, the LVOT gradient had decreased from 97 to 64 mmHg, yet the exertional chest tightness persisted, though less frequently. Similarly, the bilateral lower limb edema had improved from 2 + −3+ to 1 + −2+ and calf circumference had decreased from 27–30 cm to 26–28 cm, but had not fully resolved. Notably, the estimated PASP had paradoxically increased to 41 mmHg. If the PH were merely a passive consequence of elevated left atrial pressure from oHCM, it should have improved—not worsened—following LVOT unloading, suggesting that additional factors were sustaining the PH independently of the left ventricular outflow obstruction.

PH is a common complication of oHCM, with reported prevalence ranging from 12.6% to 51% depending on the population studied [4, 5, 6]. The present patient—an 80‐year‐old woman with markedly elevated E/e’ (21.3)—shared three of these risk factors. In a multicenter study of 70 patients with oHCM, mavacamten reduced PASP from 33 ± 10 mmHg to 29 ± 6 mmHg within a median of 12 weeks, with a more pronounced effect in patients with elevated baseline PASP [3]. While most reported studies have described a reduction in PASP following mavacamten therapy, the paradoxical rise observed in the present case, despite similarly elevated baseline PASP (35 mmHg), has received limited attention in the literature. This may reflect delayed recovery of pulmonary vascular remodeling [10] and underscores the importance of distinguishing between isolated and combined post‐capillary PH. Notably, an analysis of the FDA Adverse Event Reporting System database including 1041 adverse event reports did not identify pulmonary artery pressure elevation as a recognized adverse event signal associated with mavacamten therapy [11], supporting the interpretation that the subsequent rise in PASP reflects the underlying pulmonary vascular pathophysiology rather than a direct adverse effect of mavacamten.

Per guideline recommendations, right heart catheterization was performed and revealed an mPAP of 31 mmHg, a PCWP of 21 mmHg, and a PVR of 2.9 WU, confirming CpcPH (Group 2) with superimposed pulmonary vascular remodeling [7]. In a cohort of 162 patients with oHCM and advanced heart failure (predominantly NYHA class III/IV), 51% had PH and 34% had elevated PVR (> 3.0 WU) [6]. The present case suggests that CpcPH may also develop in patients with less advanced heart failure (NYHA class II), in whom the hemodynamic changes may be subtler and more easily overlooked. The markedly elevated E/e’ ratio of 21.3 indicated severely increased left ventricular filling pressure, which was transmitted backward to the left atrium (diameter 44 mm), the pulmonary veins, and ultimately the pulmonary arterial system [7]. Two additional factors likely accelerated pulmonary vascular remodeling: mild but chronic hypoxemia (PaO_2_ 72 mmHg) and prior iron deficiency anemia, which has been independently associated with PH [12].

The temporal sequence of echocardiographic findings (Table 1) supports this model. The initial PASP of 35 mmHg reflected the combined effect of elevated left atrial pressure, hypoxemia, and anemia, while the paradoxical rise to 41 mmHg at 2 months, despite substantial LVOT gradient reduction, is best explained by the delayed reversal of pulmonary vascular remodeling [10]. This time lag likely reflects the time needed to reverse established pulmonary vascular remodeling and should not be misinterpreted as treatment failure.

In this hemodynamic context, withholding targeted pulmonary vasodilator therapy was critical. The paradoxical rise in PASP could easily be misinterpreted as worsening PH warranting additional therapy, but current guidelines explicitly state that drugs approved for PAH are not indicated in PH‐LHD and may cause harm [7]. The elevated PCWP confirmed that the upstream driver of PH remained left heart disease, and the PVR of 2.9 WU, consistent with CpcPH, reflected pulmonary vascular remodeling that was a consequence of, not independent from, the left heart pathology.

The two main messages are: first, do not ignore a small but paradoxical PASP increase under mavacamten when symptoms and edema persist; and second, always confirm hemodynamics before starting PAH targeted drugs in suspected Group 2 PH.

In the present case, although the therapeutic decision remained unchanged—mavacamten was continued without adding pulmonary vasodilators—the invasive hemodynamic confirmation was essential in justifying this course of action. Moreover, these findings add to the limited real‐world data on mavacamten in very elderly patients with oHCM and Group 2 PH.

### 5.1 Strengths and Limitations

A key strength of this case was the systematic hemodynamic investigation of a discordant treatment response, which revealed CpcPH and directly informed the decision to withhold targeted pulmonary vasodilator therapy.

The case is limited by its single‐patient design, the absence of baseline invasive hemodynamic data, and the lack of long‐term follow‐up to confirm whether PASP declines with continued mavacamten therapy.

## 6 Conclusion

A modest, easily overlooked rise in pulmonary artery pressure after successful mavacamten therapy for oHCM may be the only clue to underlying CpcPH (Group 2) and warrants hemodynamic investigation before therapeutic decisions are made.

## Author Contributions

Mengdian Zhang: conceptualization, investigation, writing – original draft, writing – review and editing.

## Funding

The author has nothing to report.

## Ethics Statement

As a single‐case report with the patient's signed consent, no other ethical review was required.

## Consent

Written informed consent was obtained from the patient for the publication of this case report.

## Conflicts of Interest

The author declares no conflicts of interest.

## Data Availability

The data that support the findings of this study are available from the corresponding author upon reasonable request.
